# An Assessment of Success Factors and Challenges in Implementation of Electronic Medical Record System in Referral Hospital in Northern Tanzania

**DOI:** 10.24248/eahrj.v7i2.741

**Published:** 2023-11-30

**Authors:** Emanuel Q. Nuwas, Joshua G. Gidabayda, Fanuel Bellet, Godfrey Guga, Martin Matu

**Affiliations:** a Haydom Lutheran Hospital, Haydom, Manyara, Tanzania; b Eastern and Southern African Management Institute (ESAMI) Arusha, Tanzania

## Abstract

**Introduction:**

The Electronic Medical Record (EMR) has significant benefits in improving the quality of hospital services in low resources settings. Despite efforts to implement various EMRs in different health facilities, there is scarce information on the challenges and success factors regarding EMR Implementation in Regional hospitals. The aim of this study is to assess the success and challenging factors in the implementation of an electronic medical record system at the regional referral Hospital.

**Methodology:**

This was a cross-sectional design study involving qualitative and quantitative approaches that was conducted at Haydom Lutheran Hospital a Regional Referral Hospital in northern Tanzania. The semi-structured questionnaires and the Key Informant Interview Guide questions were used for quantitative and qualitative data collection respectively. The quantitative data were analyzed using Stata Version 13.0. The quantitative data was summarized using descriptive statistics. Thematic method was used to analyze the qualitative data.

**Results:**

Among 303 participants more than half were male 167(55.1%) and 119(39.3%) aged between 31 and 40 years. The nurses and medical attendants were the predominant group 188(62%). Most of the staff were on full-time employment 273(90.1%) and more than thirty percent 118(38.09%) have worked for over 10 years. The age group of between 31–60 years had a higher influence on the EMR net benefit compared to respondents aged 20 to 30 years and 60 years. The easy use, learning, usefulness, and relevance to work as well as leadership, staff involvement in processes, and use of champions were among of success factors for EMR implementation. Challenges include inadequate training, lack of funding, and inadequate IT equipment. The net benefit includes increases in efficiency in service delivery and better resource management.

**Conclusion:**

Staff involvement, use of champions and the fact that the system is easy to use contributed to the success of EMR system. In order to scale up and sustain the EMR system in hospitals, adequate funding, training as well as continuous support to all staff in the hospital is required.

## INTRODUCTION

The electronic Medical record (EMR) is important in reaching equitable and quality universal healthcare.^1^ Its use in health facilities and health systems enables understanding the disease patterns at the facility, country, and international levels to assist in planning and patient care.^2^

The uptake of EMR has been slow worldwide at rate of 12% to 18.8% in 2009 and progressively raised to around 86% in 2018 in Western countries. A significant low adoption was observed in developing counties mainly in Sub-Saharan countries.^3,4^

Globally the low adoption was associated with concerns about privacy and security, high cost of startup, workflow changes, system complexity, lack of reliability and interoperability, and resistance to change.^5^ The facilitating factors were the presence of quality of systems, use, usefulness, user perception, and user satisfaction of systems and the earned net benefit which includes automated clinical functions, practices communication, audit, and feedback reports and show a net benefit of increasing productivity, efficiency and quality data.^6,7^

In USA and China, the use of EMR had the benefits of shorter hospital stay days, lower mortality due to quicker patient treatment process, and better communication, and coordination in the provision of patient care, however, there were some concerns about efficiency, quality, and interoperability.^2, 3, 4, 8, 10^

The use of EMR in developing countries has shown significant benefit which includes, improved work process, timely secured patient data for the provision of quality patient care, improved hospital management, disease surveillance, and in research.^11–13^ The EMR user's satisfaction with system use resulted from improved patient care and increased individual staff performance.^14^ The users were also motivated to use the system due to the simplicity of system functionality, availability of user training, presence of technical support, adequate infrastructure, presence of financial support, strong leadership support, and presence of a phased implementation plan. Despite significant benefits, the slow uptake observed was found to be associated with the absence of IT experts, lack of internet connectivity, lack of electricity, power backup, lack of funding, limited computer skills, and the absence of interoperability of the system among many other factors.^11, 15, 17, 18, 19, 20^ Additionally, there were negative impacts posed by EMR implementation such as slowness in patient flow, staff frustrations, and even caused staff early retirement.^22, 24^

In Tanzania EMR introduction and implementation started in the absence of a policy framework^13, 20, 25–26^ and experienced slow progress of implementation. Currently, there are around 160 EMR Systems that are at different levels of implementation in the departments of few hospitals.^27, 28^ There has been a number of challenges encountered by health facilities in implementation processes that include poor ICT infrastructure, lack of participatory approach, lack of policy, computer skill, and security and privacy concerns.^29, 30^ These issues slowed adoption and delayed opportunities to optimize the benefit of EMR use in the provision of quality healthcare, disease surveillance, and research.

There is scarce data on the success and challenges of EMR implementation at the facility level in Tanzania. The aim of this study was to assess the success and challenging factors in the implementation of an electronic medical record system at a regional referral hospital in the Manyara region and later share the experience with the hospital and other relevant stakeholders to further improve the EMR system implementations in the hospital to optimize its benefit.

## MATERIALS AND METHODS

### Study Design

A cross-sectional study was conducted between 15th August to September 30th, 2021 and used both quantitative and qualitative approaches. The study was conducted at Haydom Lutheran Hospital in Northern-Central Tanzania. Its catchment area composed of more than 2 million population from 5 regions.

### Study Population

The target population comprised hospital leaders and staff who are implementers and users of hospital EMR excluding students and volunteers. Thus, this study targeted Physicians and Clinicians (74), Nurses and Attendants (188), Hospital managers (19), Pharmacists (10), Laboratory scientists and technicians (5) and Technical assistants (7).

### Inclusion and Exclusion Criteria

All staff working at Haydom Lutheran Hospital were eligible, and those who voluntarily consented to participate were asked to sign a written consent form. Excluded from this study were staff who did not consent, short-term visiting professionals, volunteer staff, non-medical staff and students.

### Data Collection

The quantitative data were collected using structured questionnaires which had socio-demographic information and a seven-point Likert scale to obtain the staff response on system user response. The Qualitative data was obtained from the key informant using the key informant's interview guide to get in-depth on EMR implementation processes, challenges, successes, their experience, and user experience. All key informant interviews were recorded.

### The Sample Size Estimation

Considering the proportion of staff fully utilizing EMR is 50% as reported by a study conducted in Tumbi hospital,^27^ 95% confidence interval and margin of error of 6%, the calculated sample size was 267. Taking into account non-response rate of 10%, the sample size was increased to 293. However, the response rate was 103%. That is a total of 303 individuals responded to the questionnaire. For qualitative data collection, 50% of hospital leaders and heads of departments were interviewed.

### Sampling Technique

Stratified sampling technique was employed to get the representatives from the departments and individual cadres. Health care providers were stratified into physicians and clinicians, nurses and attendants, Hospital managers, Technical assistants, Pharmacists, and Laboratory scientists and technicians. In each stratum, hospital staff were conveniently sampled. Those who were available at the time of data collection were recruited. The 11 key informants were purposively sampled for in-depth interview.

### Data Analysis and Presentation

The quantitative data analysis was done by using Stata Version 13.0. Descriptive data was analysed whereby numerical data were summarised using mean and the standard deviation, while categorical data were summarized using frequency and percentage. Analysis of variance (ANOVA) was used to determine the individual factors associated with the net benefit of EMR. *P* value of less than .05 was regarded as significant. The success factors for net benefit/EMR outcome variables were analyzed by using the Structural Equation Modelling (SEM) method to examine their strength and relationship between the variables at P<0.05. The findings were presented using tables and figures. The SEM analysis using factors analysis on 34 factors assessed the relationship among the factors and questions where the Bartlet test of sphericity was done, Eigen Values were set at >=1 were included in the next step and the loading value of 0.4 were picked.

Audio-recorded voices were transcribed word by word in the Swahili language and later translated word by word in English for analysis purposes. The notes were taken on observations and those collected during audio recordings were typed and then rechecked by the data analyst for accuracy. Thus, transcripts and notes on observation formed the data set that was analyzed using the thematic analysis method.

### Ethical Consideration

The study's ethical clearance was obtained from ESAMI Business School Ethical Review Board. The confidentiality of every participant was highly maintained by using codes. All participants signed informed consent form before collection of data.

## RESULTS

### Socio-demographic characteristics of staff involved in EMR implementation

A total of 303 participants were enrolled in this study. More than half 167(55.1%) of the participants were male and 3% were above 60 years. Of the health cadres involved, nurses and attendants composed 188(62%) followed by Clinicians/physicians 74(24.4%) Most of the staff 273(90.1%) were on full-time employment terms and 118(38.09%) had been working for over 10 years. Use of EMR system was low reported by 193(63.7%) of study participants.

The significant social demographic factors influencing EMR net benefits were age groups range of 31–40, 41–50, and 51–60 with the highest mean scores of 34.38, 34.07, and 34.79(P=0.027); respectively than staff with the age of above 60 years and 20–30years. The technical assistants, pharmacists, and Physicians/clinicians had the highest scores of 37.86, 35.00, and 34.19 respectively despite not being statistically significant (0.127).

On employment status, the staff with full-time working terms had the highest mean score of 33.86 (p=0.047) of EMR net benefit than part-time workers with a lower Mean score of 31.67 and causal workers. The staff with working experience of more than 10 years had the highest mean score of 34.37 (p=0.0007) as compared to their counterparts (Table 1).

**TABLE 1:** Socio-demographic characteristics of staff involved in EMR implementation and their Influence on the implementation of EMR (N=303)

						95% Confidence Interval for Mean				
Variables	N	Percentage (%)	Mean	Std. Deviation	Std. Error	Lower	Upper	Min	Max	P-value
Age in years										0.027
20–30	79	26.1	31.56	6.596	.742	30.08	33.03	7	42	
31–40	119	39.3	34.38	5.946	.545	33.30	35.46	14	42	
41–50	56	18.5	34.07	7.502	1.003	32.06	36.08	0	42	
51–60	42	13.9	34.69	5.559	.858	32.96	36.42	23	42	
Above 60	7	2.3	32.71	10.626	4.016	22.89	42.54	9	40	
Gender										0.245
Female	136	44.9	33.10	6.968	0.597	31.92	34.28	0	42	
Male	167	55.1	33.99	6.239	0.483	33.03	34.94	7	42	
Working Cadre										0.127
Physicians/clinicians	74	24.4	34.19	6.661	0.774	32.65	35.73	9	42	
Nurses/attendants	188	62.0	33.29	6.472	0.472	32.36	34.22	0	42	
Hospital managers	19	6.3	33.53	6.744	1.547	30.28	36.78	22	42	
Technical assistants	7	2.3	37.86	3.078	1.164	35.01	40.70	34	42	
Pharmacist	10	3.3	35.00	3.018	0.955	32.84	37.16	30	40	
Lab scientist/technicians	5	1.7	27.60	12.992	5.810	11.47	43.73	7	39	
Work status										0.047
Full time	273	90.1	33.86	6.422	0.389	33.09	34.62	0	42	
Part-time	27	8.9	31.67	7.711	1.484	28.62	34.72	7	41	
Casual	3	1.0	26.67	4.933	2.848	14.41	38.92	21	30	
Working experience years										0.007
Less than 1	39	12.9	30.54	7.152	1.145	28.22	32.86	7	41	
1 to 5	57	18.8	34.67	5.801	0.768	33.13	36.21	10	42	
6 to 10	89	29.4	33.20	6.386	0.677	31.86	34.55	14	42	
More than 10	118	38.9	34.37	6.630	0.610	33.16	35.58	0	42	
How often use EMR										0.573
Rare	193	63.7	33.84	6.663	0.480	32.89	34.79	0	42	
Often	49	16.2	32.73	6.330	0.904	30.92	34.55	9	42	
Very often	61	20.1	33.49	6.556	0.839	31.81	35.17	14	42	

The results show that most variables had high Mean Score. The variable on the useful system had the highest mean of 5.05 and was followed by the easy-to-use and learn system (Table 2).

**TABLE 2:** System Quality

Variables	Mean	Std._Dev_	Analysis
This electronic medical record system is easy to use.	4.96	.960	303
This electronic medical records system is useful	5.05	1.027	302
This electronic medical record system is easy to learn	4.85	1.066	302

On the quality of information of the EMR system, three factors had high mean namely the relevance of the system to their work (mean of 4.95), confidence in using the systems (4.90) and the systems was easy to understand (4.83). The rest of the factors had relatively lower mean score (Table 3).

**TABLE 3:** Information Quality Factors

Variables	Mean	Std._Dev_	Analysis
The information from the electronic medical system is relevant to my work	4.95	1.083	302
The information I get from this electronic medical record system is accurate	4.80	1.007	302
It is easy to understand information from this electronic medical record system	4.83	.976	301
The information in the system is presented in a useful format	4.67	1.077	301
I can retrieve in information I need easily from the system	4.75	1.103	301
Overall, I am satisfied with the electronic record system	4.77	1.115	303
When I enter data into the computer using the electronic medical record system, I feel confident about what I am doing	4.90	1.048	303
I feel comfortable to use the electronic medical record system during clinical service delivery.	4.83	1.016	299

Almost all the factors related to system quality in the EMR system support had a lower mean score (Table 4).

**TABLE 4:** Service Quality Factors

Variables	Mean	Std._Dev_	Analysis
The support services from the CareMD services provider for the system are available on time	4.40	1.188	297
The support services technical unit at the hospital for the system is usefuland helpful	4.49	1.220	300
There are services follow up by the service provider on time	4.31	1.155	299
Overall, the support services meet my needs whenever needed	4.46	1.125	300

An organizational factors analysis reveals that most of the factors had slightly higher mean score. The participants had slightly agreed that there was useful support from top management and department leaders. They also slightly agreed that there was adequate funding, adequate internet, and IT equipment (Table 5).

**TABLE 5:** Organizational Factors

Variables	Mean	Std._Dev_	Analysis
The support services from top management during the system are useful and supportive	4.56	1.185	295
The communication from top management during system implementation and usage is available and supportive	4.49	1.190	296
The supportive services from departments/leaders for the system are useful and supportive	4.57	1.118	300
The support services from the peer staff for the system are useful and supportive	4.77	.981	299
The funding to support the system is available and adequate	4.36	1.225	267
The internet and internet connectivity are available and adequate for system operation	4.34	1.254	298
The IT equipment to support the system implementation and use is available and adequate	4.29	1.300	294

### Human Factors

Most of the human factors had a lower mean score except that participants agreed on the system is useful and accepted from daily use as supporting them to system implementation. This explains that there was inadequate training and less learning time which may have leading to inadequate knowledge about the system (Table 6).

**TABLE 6:** Human Factors

Variables	Mean	Std._Dev_	Analysis
There was enough time for me to familiarise with the system during the training during the careMD implementation	4.14	1.516	300
I have access to support training	4.17	1.493	300
The training I received was adequate and relevant to how I should use the system	4.18	1.467	298
I have enough knowledge of the system	4.36	1.249	302
I have accepted the system for daily use in the car of a patient	4.79	1.124	299
I found the system useful	5.01	1.118	300

### Net Benefit/Outcome

Regarding factors on the net benefit of Implemented EMR had a higher mean score, which explains that most of the participants agreed that these are outcomes associated with implemented EMR in regional hospitals (Table 7).

**TABLE 7:** Net Benefit/Outcome

Variables	Mean	Std_.Dev_	Analysis
Using the electronic medical record system has helped me to manage patient bills, treatment, drugs, and investigations	4.94	1.190	296
Using the system has helped me to manage patient care efficiently & effectively.	4.89	1.055	301
Using the system the system has improved communication with other health service providers (e.g specialists, GPs, nurses, paramedics (pharmacy, laboratory team, and interns).	4.93	1.017	300
Using the system has facilitated me to exchange care strategies with co-workers	4.80	.964	300
Using this system has facilitated the identification of trends and patterns of diseases, resource utilization, and staff workload	4.82	1.044	298
The electronic medical record has facilitated the development of care plans in Hospitals, wards, clinics, offices, and storerooms at my workplace	4.90	1.007	296
The system gives me useful reminders that help me to identify the change of care needs for the patient in a timely manner	4.86	1.217	296

### Pattern Matrix for Factor analysis for variables

Pattern matrix where the factor analysis was performed, where seven factors and their related variables with positive factor loading were identified and noted. The factors with positive loading which means that the participants were in agreement that those variables were positively influencing the EMR implementations. Those variables are as follows. These Factors were named as Factors 1–7. Factor 1 was the usefulness and functionality of the EMR.

Factor 2: There was Inadequate basic and supporting training, and insufficient time spent during training on EMR as the Participants were not in agreement.

The other factors were Factor 3 which was the Availability and accessibility of the EMR technical support as the Participants were in agreement. Factor 4 where the participant's Comfortability with the system, the format of the information, and easiness retrieval of the information from the system where participants agreed that they were influencing the EMR Implementation.

Factor 5 includes the unavailability of the equipment, funding, inadequate infrastructure, and lack of communication and support from leaders during EMR implementation.

Factor 6 was that there was Simplicity of learning the EMR, understating and using the system by staff while Factor 7 shows that The Lack of understanding of the application of the EMR on improving patient care was not influencing the EMR Implementations.

### The Structural Equational Model Analysis for EMR implementation in a regional referral hospital

**FIGURE 1:**
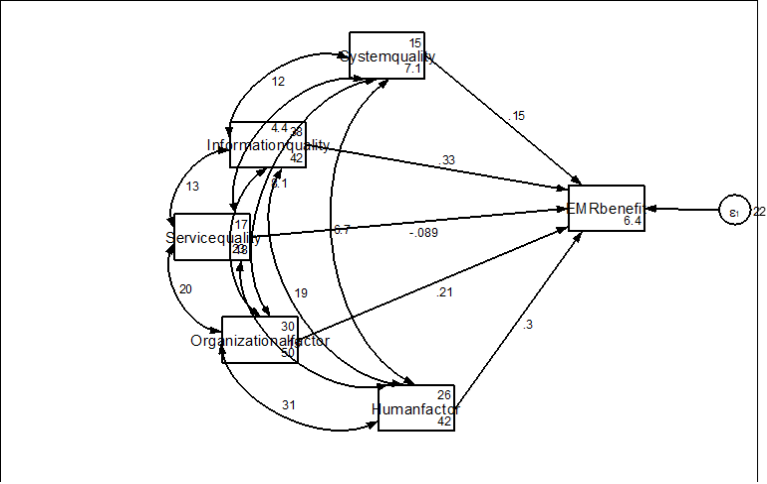
Structural Equational Modeling for the Factors Associated with EMR Effective Implementation or Net Benefit

From the Structural Equational Analysis model, our observed dependent variable was EMR benefits Exogenous variables with Fitting target model (Iteration 0: log-likelihood = −5160.4837; Iteration 1: log-likelihood was −5160.4837); our independent variables were system quality, information quality, service quality, organizational and human factors. The results from SEM indicated the regression coefficients indicating that technical factors (system quality and information quality), organizational and human factors had a positive contribution to EMR implementation with regression coefficients of 0.15, 0.33, 0.21, and 0.30 respectively. However, the significance test indicated that information quality (p<0.001), organizational factors (p=0.001), and human factors (p<0.001) were significant predictors of the net benefit of EMR at regional referral hospitals. Therefore, system quality and service quality were statistically significant variables to explain EMR net benefit. There was covariance observed from each independent variable in the model with p<0.05 (Figure 1 and Table 8).

**TABLE 8:** The SEM Analysis for the Success fFctors of EMR Implementation in a Regional Level Referral hospital

Structural	Coef.	Std. Err.	[95% Conf. Interval]		P-value
System quality	0.150522	0.1471767	−0.137939	0.438983	0.306
Information quality	0.3259366	0.0630612	0.2023389	0.4495343	<0.001
Service quality	−0.0888568	0.0887879	−0.2628778	0.0851642	0.317
Organizational factor	0.2056055	0.0604417	0.0871419	0.324069	0.001
Human factor	0.2967183	0.0576305	0.1837645	0.409672	<0.001
_cons	6.356285	1.757533	2.911584	9.800986	<0.001
Mean (System quality)	14.82178	.1530434	14.52182	15.12174	<0.001
Mean (Information quality)	38.32673	.3743897	37.59294	39.06052	<0.001
Mean (Service quality)	17.42904	.2427981	16.95317	17.90492	<0.001
Mean (Human factor)	26.36634	.3737737	25.63375	27.09892	<0.001
Var (e.EMR benefit)	22.14952	1.799526	18.889	25.97286	–
Var (System quality)	7.096951	.5765879	6.052244	8.321991	–
Var (Information quality)	42.4708	3.450517	36.21889	49.8019	–
Var (Service quality)	17.86213	1.451199	15.23273	20.9454	–
Var (Organizational factor)	49.76678	4.043274	42.44085	58.35726	–
Var (Human factor)	42.33114	3.43917	36.09978	49.63813	–
Cov (System quality, Information quality)	12.45757	1.227577	10.05156	14.86358	<0.001
Cov (System quality, Service quality)	4.353691	.6934899	2.994476	5.712906	<0.001
Cov (System quality, Organizational factor)	8.125413	1.176243	5.820019	10.43081	<0.001
Cov (System quality, Human factor)	6.728654	1.068136	4.635147	8.822161	<0.001
Cov (Information quality, Service quality)	12.6585	1.741416	9.245385	16.07161	<0.001
Cov (Infonnation quality, Organizational factor)	22.61056	2.943288	16.84182	28.3793	<0.001
Cov (Information quality, Human factor)	18.63278	2.660689	13.41793	23.84764	<0.001
Cov (Service quality, Organizational factor)	19.93949	2.060571	15.90085	23.97814	<0.001
Cov (Service quality, Human factor)	14.98474	1.799034	11.4587	18.51078	<0.001
Cov (Organizational factor, Human factor)	30.86799	3.177643	24.63992	37.09605	<0.001

### The Qualitative Data Findings on Success, Challenges Factors, and Net Benefits in EMR Implementation at the Regional Level Referral hospital

A total of 11 participants from study participants were involved in the study where 9 were male and 2 females. The investigator carried out 11 key informants' interviews. Thematic analysis was done whereby the voice recorded was familiarized and transcribed to form the codes, which led to the formation of subthemes and 3 major themes (outcome of EMR, success factors, and challenges for implementations of EMR at regional referral hospitals) (Table 9–11).

**TABLE 9:** Success Factors Enabling the Implementation of EMR at Regional Referral Hospital

Themes	Sub-themes	Coding and key responses
Success Factors enabling the implementation of EMR at RRHs	Staff management and supervision and use of	HD102…me As the manager in the ward for staff & and resources, I supervised the use of systems and to use in my daily care of patients … HD104…. I manage the training and support the staff in using the System, where I also use it personally ….
	High acceptance, satisfaction, and usage of systems among the users	HD 101…I will say maybe most about 80–90% of my staff have accepted and satisfied with the use of systems… HD 103… they like as it has made life easy in this very difficult work …. HD 106…the staff at my department are almost fully satisfied and are effectively using the systems during the care delivery, and they like….
	Adequate support	HD 105…like whenever we need help the IT team they come to fix it…. HD 107… we contact him very often and he answers any questions and helps with any troubleshooting offsite electronically… and it works so … HD 102… I saw the provider coming every here and then but I think he answers a lot from IT personnel, so he is not in contact with us…
	The staff involvement and precipitation Training champions Internet and electricity stability	HD 101 …. I remember we frequently share information and education to staff in every meeting and prepared them for the systems …. HD 101…. we did the training but we identified a staff who were champions and underwent training and later they started training their peer staff in their wards I liked it as it helped to make more staff trained and use the system….. HD 103…. having a good bandwidth that is connected in 24 hours made the staff like it… HD 101…Our hospital has standby generators which have ensured full-time electricity so we had an interruption in few times…..

Key: HD – Head of department

The challenges observed were inadequate funding, absence of policy and strategies to support the EMR project, inadequate system user training, and change resistance from some staff members. The other challenges see (Table 10).

**TABLE 10:** Challenges for Implementations of EMR

Themes	Sub-themes	Initial Coding
Challenges for implementations of EMR	Inadequate Funding	HD 108…. we are not sure if there was a set funding for the project…. HD 110…we had some budget for IT improvement but not specific and this is the fund that was used to support the work…. HD 105….am not sure if there was set budget for that purpose …. HD 109…. we did not have any funding set aside for this system…
	Poor policy and strategies	HD 103….am not sure if there was any policy or strategy in place maybe the management knows much about this… HD 101…. yes, it was a challenge before we had a policy for the implementation of EMR, and this was a challenge for EMR implementation at our facility. Now, we have a policy in place that we developed later when we realized that we needed it to guide the process and safeguard the use, which is what we called ICT policy that we have now and using it…
	Inadequate training to few staff	HD 107…. honestly the training was given to staff from various departments in groups but they were only a few selected and most did not get the proper training so other were trained by their friends in the ward but was not enough to understand in time…. HD103……in short, the training is given was to only a few selected staff from our wards who were champions and they really helped to train and motivate others in the ward however I think it was not enough as it was short duration… HD 109…. the organized training was relevant, but it was in few hours a day and they did in few days and of course, it was given to few staff… HD 111 …it was in the beginning I don't remember if there was another session in between…
	Change resistance Inadequate training Inadequate Computer illiteracy System blackout Inadequate IT equipmer Donor dependency on Equipment	HD103…I remember there were some few staff just resisting the use of the electronic system and mobilizing others to resist as well…. HD106. what I can say is that there was a number of staff who were computer illiteracy … HD 101…. the system cutting off anytime and cause al lot of disturbance to patients and staff sometimes IT people will help us after some time it happens…. HD105…..Sometimes at sites, the computers are not work ing, or they are out of order this has been one of discouraging factors … HD110……we depend on the donor for IT equipment so mostly there is equipment not working inward or doctor room like a computer or its stacking as it's so old…

Key: HD – Head of department

The following outputs were reported as benefits of the EMR system in regional hospitals which are availability of timely report, improved resource management, and increased efficiency in service delivery (Table 11).

**TABLE 11:** Experienced Outcome/Benefits of EMR

Themes	Sub-themes	Initial Coding
Experienced outcome/benefits of EMR	Timely and quality reports. The improved management of humans' resources, financial resources Efficiency in service delivery	HD 101…it is now easy for me to quickly etrieve the report dad then I gather them and prepare for decisions making at any level in the hospital… HD 105…I can follow while here in the office the following materials in the store …. HD 101…I must admit that now I can follow the hospital collections at collections points and see the report of any duration whenever I need to review of prepare for the meeting so am happy… HD 107…. As department leader, I can follow the work of my fellow staff on the computer, and sometimes I can call if I need to communicate something…..
	Increased efficiency Good control of human, financial, and inventory resource management Timely and quality of reports Staff satisfaction	HD109….I must say a5 a leader this has made it easy to monitor the work as I can sit here and follow the work in the wards and see who is doing what and where… HD101…I can retrieve the report when even I need it and see my staff performance any time … HD105…I can review the patient file whenever there is a complaint and that helps me make good decisions… HD110 ….…compared to when it was paper from the file you must suffer reading different notes with all handwriting…s you can imagine…. HD107…. I must tell you it was cumbersome and sometimes the documents get lost, and you can't retrieve them…

Key: HD – Head of department

### The Outcomes Net Benefit of Fully Implemented EMR in the Regional Referral Hospital Services

The mean score above 4 was considered to indicate good strengths of agreement with the identified net benefits from EMR and was observed as the positive outcome or net benefit from using EMR. All indicated high strengths of agreement with a mean score >4 (Table 12).

**TABLE 12:** The Outcomes Net Benefit of Fully Implemented EMR in the Regional Referral Hospital Services

Outcome of EMR	SD/D	N	SA/A	Mean	95%CI
Easy to manage patient's bills, treatment, drugs, and investigations.	19(6.3)	23(7.6)	261(86.1)	4.82	4.66 – 4.98
Helps to manage patient care efficiently and effectively.	12(4.0)	18(5.9)	273(90.1)	4.85	4.73 – 4.97
Improves communication with other health service providers e.g. specialists, GPs, Nurses, paramedics pharmacy, laboratory team, and Interns.	9(3.0)	20(6.6)	274(90.4)	4.88	4.76 – 5.00
Facilitate the exchange of care strategies with co-workers.	9(3.0)	18(5.9)	276(91.1)	4.75	4.63 – 4.87
Facilitates the identification of trends and patterns of diseases, resource utilization, and staff workload.	14(4.6)	24(7.9)	265(87.5)	4.74	4.59 – 4.88
Facilitate the development of care plans in hospitals, ward, clinics, office, and storerooms, at my workplace.	13(4.3)	22(7.3)	268(88.4)	4.79	4.64 – 4.92
Useful to identify the change of care needs for a patient in a timely manner.	24(7.9)	22(7.3)	257(84.8)	4.75	4.1 – 4.91

## DISCUSSION

In this study, it is found that more than half of the respondents were males compared to observed female dominance and the majority were age group of 31–40 and 41–50 years this is like finding in a study done in Ethiopia.^34^ These age groups were significantly influencing the EMR Net Benefit this is likely due to being the long-serving staff and having learned the systems, and they may have adequate computer literacy. Furthermore, the study found that the young age groups between 20–30 and older age staff over 60 years did not have influence and this could be due to the reason that the younger age staff were newly employed, and old age were having computer illiteracy. This illiteracy in computer use was found in the qualitative analysis as one of the challenges among hospital staff and may have been contributed to observed inadequate training provided.

There is the importance of continuous staff training as observed in one systematic review study that there is a need for effective staff to be fully understanding the system as emphasized in a study done in the USA.^35^ Staff training is even more important in a lower-resource setting with limited computer literacy. This is key as computer knowledge and literacy are associated with high adoption of EMR systems as reported in a study done in Nigeria.^36^ This study had a similar finding as the quantitative analysis shows that the staff was not in agreement that there was adequate training and enough time to familiarize themselves with the system.

In this study, the nurses and medical attendants were a larger group composing (62%) being similar findings as reported in the Safdari study ^34^ which has (33 %) the same findings as in other studies where nurses are dominant staff in many health facilities.^34^ The Doctors and clinicians were 24.4% which is similar to findings in the following studies done in Iran and Ethiopia.^34,36^ In most health facilities, nurses and attendants outnumber the clinicians, so the nurses are likely to adopt system change.

The study found that the majority of staff were on full-time employment terms, and most have served for over ten years followed by a group who has served between 6 and 10 years. The staff who have worked for over ten years and those on a full-time basis were significantly influencers of EMR outcomes while staff who has served in a short period of between 1 to 5 years were not influencing the EMR net benefit. The above difference implies that the longer the staff work in the hospital, the better understanding of healthcare provision in different systems like EMR.

Regarding the use of EMR, this study found that most of the staff were rarely using the system compared to the small proportion who often used the system. This could be due to the fact that most of the staff proportions are nurses and medical attendants who are using the system only for medication and also their nature of work where they work in shifts and other duties that do not need the use of the system. Most Physicians Clinicians and paramedics are likely the staff who use the system very often, but they are in small proportions. This is a key negative finding that requires to be further evaluation as this may be a risk factor for the sustainability of the system and the quality of the information in the system which may affect the quality of reports and other system outputs. Additionally, clinicians are the ones who document most during patient care.

The factors which were found to be influencing the successful implementation of EMR in hospitals were easy to learn the system, easy to use, and the staff found the system useful. These findings are similar compared to the results of other studies which reported that ease of use, user-friendly, and usability were the most contributory success factors as well the under-technical factors.^34,36^ ^37^

The service quality-related factors were found to be influencing the successful implementations of EMR these factors include the adequacy of support service from the system provider despite that the support was frequently through online support according to qualitative data. The presence of adequate support from technical units at the hospital. This is similar to findings reported in a study done in Ethiopia.^36^ Meanwhile, the support from the department leaders was not contributing as staff disagreed as it had a Negative loading in factors analysis. This may be explained by the level of support the head of the departments were able to accord to their fellow staff during implementation was not enough due to the fact that they were learners at the same time.

Regarding the information quality factors, the study found that the staff was satisfied with the systems, and they were confident when they are using it, they further agreed that they can easily retrieve information from the system and this is similar findings as in systems quality regarding the easiness of use of the system. The data shows that the system is in a useful format and staff feels comfortable using it for clinical service delivery. All the above factors had high Mean in the Descriptive analysis of individual factors and positive loading in Factor analysis. The above findings were similar to the reported high acceptance of systems and higher staff satisfaction with the usage of systems in qualitative analysis. Since the system is well accepted by staff, it is very clear that it can be applied in all aspects of health care delivery.

The factors like the relevance of the system to their work though it had a high mean value (4.95) in descriptive analysis and the accuracy of the system were not found to be contributing during the analysis of both qualitative and quantitative data. This will require in-depth analysis to understand its irrelevancy to staff work and accuracy level as the system may be lacking some functionality.

In human factors, the study found that most of these were not contributing to success factors in the implementations of EMR. In both qualitative and quantitative analysis similar findings were observed where the qualitative analysis revealed that the training was given to a few staff who were champions. Then they further trained their peer staff in the wards and this seems that the training was inadequate and there was no evidence that there was support training during the use of the system. These findings may be resulting from the provision of inadequate basic and supportive training, and less learning time for staff to familiarize themselves with systems all may have led to inadequate knowledge of the staff on the system as all factors had negative loading in factors analysis and lower Mean in Descriptive analysis. This may hamper the sustainability of the EMR system and the quality of its output if these challenges will not be addressed in a strategic way. The Importance of adequate training and knowledge of the staff is critical to the success of system implementation^37^ and improving skill, and awareness of EMR among health professionals improves EMR performance as found in a study done in Ethiopia.^33^ ^38^ The lack of proper staff training will lead to slower adoption of EMR and hence may lead to failure of the system. As the EMR is geared to completely replace conventional paper medical records then there must be sustainable training of health workers in all cadres.

Of the organization factors, the study found most were not contributing as success factors in EMR implementations in this hospital. This finding is contrary to the finding in SEM analysis, which showed that the organizational factors were statistically significant (p-value of (0.001). This may need further analysis which is beyond the scope of this study.

These findings on Organizational factors are evidenced by most having lower mean values in the descriptive analysis and negative loading in the Factors analysis. These factors are translated as there was inadequate IT equipment, lack of Internet connectivity, inadequate funding, and less support from top management and department leaders during the system implementation. In qualitative analysis, this study had the same findings, and in addition that there was no strategy and policy in place before the kickoff of the implementation of the system. These findings are different compared to the reported findings in a study done by Safdari which reported that the organizational factors, especially on the stability of management, were rated high for successful factors for EMR implementations.^34^ Among the management, factors is the Presence of policies to support the EMR implementation in health facilities and this was found in a study done by KCMC in Tanzania which noted that the lack of policies and standard approaches contributed to the failure of EMR system implementation in low resource settings.^29^ This could be the case in this study that there was no policy to support the project as observed in the qualitative part of the study despite that it was developed later stage.

Furthermore, on organizational factors, the qualitative part has observed that there was no clear funding allocation specifically for the implementation of EMR which is similar to findings that there is the importance of the availability of funding in facilitating the MER implementation in health facilities as reported in a study done in Ethiopia which found the EMR performance requires the organizational management to set resources and allocate to facilitate the process in a health facility. ^38^ The funding will enable the availability of proper IT equipment as they are key to improving EMR performance as reported in a study done in Ethiopia.^38^ The inadequacy and unavailability of such equipment may lead to failure of EMR implementation in a health facility as observed in a study done at KCMC which concluded that poor IT infrastructure was among the factors that caused the failure of EMR implementation in the low resource settings.^29^

As evidenced from the qualitative findings, it seems EMR implementations at this hospital did not have a project management plan contrary to recommendations from studies that a project management approach would have made it more successful.^34^ ^37^ This may be done through the early creation of a road map, Project Goal setting, and training of staff and gaining the knowledge and skill required.

The qualitative analysis of our study observed that one of the success factors was staff involvement and participation in a project which have a significant positive influence on EMR project implementations as reported in a study done in Sri Lanka^39^ as well as finding that lack of participatory approach among the staff at the facility was the reason for the unsuccessful implementation of the EMR in health facilities in the low resource settings.^29^

In the assessment of the challenges of EMR implementation in regional hospitals from the qualitative data, the study has observed that there are several challenges in EMR implementation in hospitals in low-resource settings. These challenges include an unstructured implementation plan. These challenges are found in both qualitative and quantitative analysis of this study which includes the absence of a proper project management plan and strategy, lack of funding to support the system implementation, inadequate IT equipment, and donor dependency on ICT equipment support with second-hand equipment as found in qualitative analysis. These are similar findings as observed in a study done in KCMC.^29^ These challenges may likely jeopardize the implementation and sustainability of the EMR system in hospitals.

Regarding the net benefits of EMR from the study through qualitative analysis, it is found that quality reports, improved human, inventory, and financial resource management, and increased efficiency in service delivery and its operations. However, these Findings were drawn from Key informants who were the leaders and ward managers. This is similar to findings observed in a systemic review study done in Asia^2^ which reported similar findings that the benefit of helping in the coordination of work, predicting the pattern of diseases, and helping decisions making process from patients' level to managerial levels based on reports produced from the EMR system use.^2,32^

The quantitative analysis has found that the following factors like easy management of patient's bills, treatment, drugs, and investigations; Better management of patient care efficiently and effectively; improved communication with other health service providers; facilitation of the exchange of care strategies with co-workers; the identification of trends and patterns of diseases, the resources' utilization, and staff workload; improves care plans; and useful to identify the change of care needs for a patient in a timely manner. All those factors had negative loading in factors analysis despite most factors having a higher mean value in descriptive analysis. This could be related to inadequate knowledge by staff on the system because of inadequate training, posing a partial understanding of the system by many of the staff.

## CONCLUSIONS AND RECOMMENDATION

The EMR system's easy use, easy learning, usefulness of systems, staff involvement, and use champions were success factors. There is a need to scale-up and sustain the EMR system in hospitals through adequate funding, training as well as continuous minimal support to all staff in the hospital. The Multicenter study is required to learn further about the matter.

## References

[1] The World Health Organization's (WHO) draft 2019 four-year global digital health strategy. Published April 3, 2021. Accessed April 3, 2021.

[2] Dornan L, Pinyopornpanish K, Jiraporncharoen W, Hashmi A, Dejkriengkraikul N, Angkurawaranon C. Utilisation of Electronic Health Records for Public Health in Asia: A Review of Success Factors and Potential Challenges. BioMed Research International. 2019;2019. doi:10.1155/2019/7341841 PMC6644215 31360723

[3] Liang J, Li Y, Zhang Z, et al. Adoption of Electronic Health Records (EHRs) in China During the Past 10 Years: Consecutive Survey Data Analysis and Comparison of Sino-American Challenges and Experiences. Journal of Medical Internet Research. 2021;23(2):e24813. doi:10.2196/24813 33599615 PMC7932845

[4] Kharrazi H, Gonzalez CP, Lowe KB, Huerta TR, Ford EW. Forecasting the Maturation of Electronic Health Record Functions Among US Hospitals: Retrospective Analysis and Predictive Model. J Med Internet Res. 2018 Aug 7;20(8):e10458. doi: 10.2196/10458. PMID: 30087090; PMCID: PMC6104443. 30087090 PMC6104443

[5] Dutta B, Hwang HG. The adoption of electronic medical record by physicians: A PRISMA-compliant systematic review. Medicine. 2020;99(8):e19290. doi:10.1097/MD.0000000000019290 32080145 PMC7034652

[6] Kruse CS, Stein A, Thomas H, Kaur H. The use of Electronic Health Records to Support Population Health: A Systematic Review of the Literature. J Med Syst. 2018;42(11):214. doi:10.1007/s10916-018-1075-6 30269237 PMC6182727

[7] O'Donnell A, Kaner E, Shaw C, Haighton C. Primary care physicians' attitudes to the adoption of electronic medical records: a systematic review and evidence synthesis using the clinical adoption framework. BMC Medical Informatics and Decision Making. 2018;18(1):101. doi:10.1186/s12911-018-0703-x 30424758 PMC6234586

[8] Lee J, Kuo YF, Goodwin JS. The effect of electronic medical record adoption on outcomes in US hospitals. BMC health services research. 2013;13(1):1–7. 23375071 10.1186/1472-6963-13-39 PMC3568047

[9] Adler-Milstein J, DesRoches CM, Kralovec P, et al. Electronic Health Record Adoption In US Hospitals: Progress Continues, But Challenges Persist. Health Affairs. 2015;34(12):2174–2180. doi:10.1377/hlthaff.2015.0992 26561387

[10] Kruse CS, Kothman K, Anerobi K, Abanaka L. Adoption Factors of the Electronic Health Record: A Systematic Review. JMIR Med Inform. 2016;4(2):e19. doi:10.2196/medinform.5525 27251559 PMC4909978

[11] Oyugi B, Makunja S, Kabuti W, et al. Improving the management of hypertension and diabetes: An implementation evaluation of an electronic medical record system in Nairobi County, Kenya. International Journal of Medical Informatics. 2020;141:104220. doi:10.1016/j.ijmedinf.2020.104220 32622341

[12] Tan SESO, Ishak NN, Yusoff NM. Prevalence of Anaemia in Children Treated in Kepala Batas, Penang. Malaysian Journal of Paediatrics and Child Health. 2020;26(2):35–50.

[13] Rutatola EP, Yonah ZO, Nyambo DG, Mchau GJ, Musabila AK. A Framework for Timely and More Informative Epidemic Diseases Surveillance: The Case of Tanzania. :16.

[14] Alsohime F, Temsah MH, Al-Eyadhy A, et al. Satisfaction and perceived usefulness with newly-implemented Electronic Health Records System among pediatricians at a university hospital. Computer methods and programs in biomedicine. 2019;169:51–57. 30638591 10.1016/j.cmpb.2018.12.026

[15] Butt FS, Mahum R, Zia A, Nawab S, Shad SA. BARRIERS IN IMPLEMENTATION OF ELECTRONIC MEDICAL RECORDS IN PAKISTAN. :6.

[16] Mashoka RJ, Murray B, George U, et al. Implementation of electronic medical records at an Emergency Medicine Department in Tanzania: The information technology perspective. African Journal of Emergency Medicine. 2019;9(4):165–171. doi:10.1016/j.afjem.2019.07.002 31890478 PMC6933271

[17] Gyamfi A. Use of electronic medical records in emergency care at Komfo Anokye Teaching Hospital in Kumasi, Ghana. 2016.

[18] Hamad WB. CURRENT Position and Challenges of E-health in Tanzania: A review of literature. 2019;7(9):13.

[19] Odekunle FF, Odekunle RO, Shankar S. Why sub-Saharan Africa lags in electronic health record adoption and possible strategies to increase its adoption in this region. Int J Health Sci (Qassim). 2017;11(4):59–64. https://www.ncbi.nlm.nih.gov/pmc/articles/PMC5654179/. Accessed July 14, 2021. PMC5654179 29085270

[20] Kavuma M. The Usability of Electronic Medical Record Systems Implemented in Sub-Saharan Africa: A Literature Review of the Evidence. JMIR Human Factors. 2019;6(1):e9317. doi:10.2196/humanfactors.9317 30801251 PMC6409508

[21] Ngugi PN, Babic A, Were MC. Users' Perception on Factors Contributing to Electronic Medical Records Systems Use: A Focus Group Discussion Study in Healthcare Facilities Setting in Kenya. In Review; 2021. doi:10.21203/rs.3.rs-331806/v1 PMC8710176 34955098

[22] Scott IA, Sullivan C, Staib A. Going digital: a checklist in preparing for hospital-wide electronic medical record implementation and digital transformation. Australian Health Review. 2019;43(3):302–313. 29792259 10.1071/AH17153

[23] Balugaba BE, Ruttoh SK, Ekirapa EK, Siika AA, Were MC. Cost Analysis of an Electronic Medical Record System at an Urban Clinic in Kampala, Uganda. Journal of Health Informatics in Africa. 2019;6(2):84–89.

[24] Crowson MG, Vail C, Eapen RJ. Influence of electronic medical record implementation on provider retirement at a major academic medical centre. Journal of evaluation in clinical practice. 2016;22(2):222–226. 26395432 10.1111/jep.12458

[25] Watts G. The Tanzanian digital health agenda. The Lancet Digital Health. 2020;2(2):e62–e63. doi:10.1016/S2589-7500(20)30005-4

[26] Macha T. Tanzania Digital Health Strategy. :18.

[27] Thadeus WK, Mushi LD. Health care professional knowledge and attitude towards the use of digital technologies in provision of maternal health services at Tumbi regional referral hospital in Tanzania. Journal of Medical Research and Innovation. 2021;5(1):e000233–e000233.

[28] Kombe C, Sam A, Ally M, Finne A. Blockchain Technology in Sub-Saharan Africa: Where does it fit in Healthcare Systems: A case of Tanzania. Journal of Health Informatics in Developing Countries. 2019;13(2).

[29] Mtebe J, Nakaka R. Assessing Electronic Medical Record System Implementation at Kilimanjaro Christian Medical Center, Tanzania. 2018;12.

[30] Nehemiah L, Mdegela L. Towards EHR Interoperability in Tanzania Hospitals : Issues, Challenges and Opportunities. International Journal of Computer Science, Engineering and Applications. 2014;4. doi:10.5121/ijcsea.2014.4404

[31] Mæstad O, Mwisongo A. Haydom Lutheran Hospital - Final Project Review. Chr. Michelsen Institute; 2009. https://open.cmi.no/cmi-xmlui/handle/11250/2436075. Accessed May 8, 2022.

[32] The DeLone and McLean Model of Information Systems Success: A Ten-Year Update. Journal of Management Information Systems. 2003;19(4):9–30. doi:10.1080/07421222.2003.11045748

[33] Yusof M Mohd, Paul RJ, Stergioulas LK. Towards a Framework for Health Information Systems Evaluation. In: Proceedings of the 39th Annual Hawaii International Conference on System Sciences (HICSS'06). Vol 5. ; 2006:95a–95a. doi:10.1109/HICSS.2006.491

[34] Safdari R, Ghazisaeidi M, Jebraeily M. Electronic Health Records: Critical Success Factors in Implementation. Acta Inform Med. 2015;23(2):102–104. doi:10.5455/aim.2015.23.102-104 26005276 PMC4430004

[35] Tabner J, Zhao F, Pavel N, Kincaid K, Murphy C. Enough or Too Much in EMR Training and Education? In: Kurosu M, ed. Human-Computer Interaction. User Interface Design, Development and Multimodality. Lecture Notes in Computer Science. Cham: Springer International Publishing; 2017:234–244. doi:10.1007/978-3-319-58071-5_18

[36] Tilahun B, Fritz F. Comprehensive evaluation of electronic medical record system use and user satisfaction at five low-resource setting hospitals in Ethiopia. JMIR medical informatics. 2015;3(2):e4106. 10.2196/medinform.4106 PMC4460264 26007237

[37] Sidek YH, Martins JT. Perceived critical success factors of electronic health record system implementation in a dental clinic context: An organisational management perspective. Int J Med Inform. 2017;107:88–100. doi:10.1016/j.ijmedinf.2017.08.007 29029696

[38] Yehualashet G, Asemahagn M, Tilahun B. The Attitude towards and Use of Electronic Medical Record System by Health Professionals at a Referral Hospital in Northern Ethiopia: Cross-Sectional Study. Journal of Health Informatics in Africa. 2015;3(1). doi:10.12856/JHIA-2015-v3-i1-124

[39] Narattharaksa K, Speece M, Newton C, Bulyalert D. Key success factors behind electronic medical record adoption in Thailand. Journal of Health Organization and Management. 2016;30:985–1008. doi:10.1108/JHOM-10-2014-0180 27681029

